# A qualitative study of infectious diseases fellowships in Japan

**DOI:** 10.5116/ijme.56b5.010c

**Published:** 2016-02-21

**Authors:** Kentaro Iwata, Asako Doi

**Affiliations:** 1 Division of Infectious Disease, Kobe University Hospital, Japan; 2 Division of Infectious Diseases, Kobe City Medical Center General Hospital, Japan

**Keywords:** Specialty training, infectious diseases, qualitative study, japan

## Abstract

**Objectives:**

The purpose of this research is to elucidate the actual status of Infectious Diseases (ID) Fellowship programs in Japan to improve them further.

**Methods:**

We conducted qualitative interviews with infectious diseases fellows and his/her faculty consultants from 10 institutions providing ID Fellowships in Japan. We qualitatively analysed the data to delineate the actual status of each program and the fellowship program policies overall, and to identify measures for further improvement.

**Results:**

The interviews revealed that there are largely two kinds of ID fellowships; ID programs entirely devoting full time to infectious diseases, and programs that are subordinate concepts of other subspecialties, where only a portion of hours were devoted to ID. Some institutions did not even have an ID department. Time spent by the faculty consultants on fellows also varied among programs. The desire for improvement also varied among interviewees; some being happy with the current system while others demanded radical reform.

**Conclusions:**

Even though there are many ID fellowship programs in Japan, the content, quality, and concepts apparently vary among programs. The perceptions by interviewees on the educational system differed, depending on the standpoints they have on ID physicians. There probably needs to be a coherency in the provision of ID fellowship programs so that fellows acquire competency in the subspecialty with sufficient expertise to act as independent ID specialists. Further studies are necessary for the improvement of ID subspecialty training in Japan.

## Introduction

Infectious Disease (ID) is an important subspecialty in medicine.^1^ Studies have addressed this topic,^2^-^4^ but few addressed the issue of program structure with adequate curriculum development in this field.

Board certification in Infectious Diseases in Japan is provided by the Japanese Association for Infectious Diseases (JAID). JAID has mandated that those who wish to become infectious diseases specialists undertake 3 years of postgraduate training at accredited institutions since 2007.^5^ However, few studies were conducted to investigate the actual status and quality of this specialty training in Japan. Although JAID provides a fellowship curriculum, it is actually a list of subjects, like the Table of Contents of textbooks, and does not provide an actual syllabus, which fellows are expected to undertake.^6^

Our previous quantitative study, conducted through a questionnaire sent to each teaching facility in Japan, found that less than half of teaching hospitals for ID physicians in Japan actually had ID fellow(s) in action. In addition, only about half of these institutions had a functioning ID fellowship program.^7^ Even though there are many ID fellowship programs in Japan on paper, many of them are not functioning enough. The secondary question we encountered after this survey was the following: what were the remaining acting ID fellowship programs like in Japan, which had never been investigated in detail?

To elucidate the actual status of Infectious Diseases Fellowship programs in Japan, their strong points and shortcomings, and to identify measures to improve them further from the viewpoints of both faculties and fellows, we conducted a qualitative study by interviewing those fellows and faculty consultants. By the same method, we also investigated the perceptions of both fellows and faculty consultants regarding their views on ID fellowship policies implemented by JAID.

## Methods

### Design

This qualitative study was conducted using semi-structured interviews with infectious diseases fellows and faculty consultants (Appendix). A modified Grounded Theory approach was taken. We extracted data through interviews, coded based on such data, categorized them into theory, a variation of grounded theory approach, but not necessarily adopted in ways originally described by either Corbin or Strauss.^8^^,^^9^

### Participant recruitment and sampling

Board certification in Infectious Diseases in Japan is provided by JAID. JAID has mandated that those who wish to become infectious diseases specialists undertake 3 years of postgraduate training at accredited institutions. To be certified as an accredited institution, there must be at least one "Shidoi", which literally means "teaching doctor" in Japanese, and is a person who is at least 5 years post ID specialist certification.5 JAID also created "collaborative institutions" on top of existing accredited institutions to increase the fellowship programs, which do not require the presence of Shidoi.^5^ As of March 2013 there were 250 accredited institutions and 97 collaborative institutions. We randomly selected a total of 10 institutions using a table of random numbers, from both accredited and “collaborative institutions”, certified by JAID. We requested the program director interviews with an ID fellow and a faculty consultant. If approved, we set the schedule and conducted interviews over the telephone. If the interview was declined by the program director, we repeated the selection of institutions randomly, until the agreement from 10 institutions was reached. Data collection was mainly conducted in March 2013, but one interview with a faculty was postponed until December 2013 due to scheduling problems. One of authors (KI), who was trained in qualitative research, interviewed all the persons who volunteered to participate. One participant declined the interview over the telephone and rather requested discussion by e-mail due to unavoidable conditions. We later decided not to include this interview in this study because it was out of our methods, resulting in the analyses of 19 interviews in total in the study.

The institutions consisted of 7 accredited institutions and 3 collaborative institutions. One collaborative institution, which was randomly selected, declined to participate in this study and another collaborative institution, which agreed to participate, was again randomly selected.

Each institution was coded both for faculty consultant and fellows by number randomly to ensure their confidentiality, so each number does not necessarily correspond to a given institution. For example, [consultant 1] and [fellow 1] may or may not belong to the same institution.

### Consent and data collection

 We obtained informed consent verbally over the telephone. After the participants’ consent, we conducted semi-structured interviews in Japanese. Interviews typically lasted approximately 20 to 30 minutes. All interviews were digitally recorded using a digital voice recorder, and stored as electronic wave files on a computer.

### Data analysis

The recorded data were transcribed verbatim. Based on multiple readings of transcripts, themes addressing the research purposes were iteratively identified by the analysis team (KI and DA). We extracted comments by interviewees as data, coded based on generated hypotheses and concepts, and grouped into several categories, which we elaborated through the reading. Developed categories were reviewed by all authors iteratively in depth to ensure rigor, to the point when all categories were well developed and further data gathering and analysis added little new to the conceptualization (theoretical saturation). Finally, remarks by the participants included in the manuscript were translated into English language. We used OmniOutliner for Mac 4.0.1 (The Omni Group) for data coding and categorization, but did not use applications developed specifically for qualitative research.

### Ethical considerations

To respect ethical considerations, the participants were assured that all the information would remain confidential, and after implementation, all the audio files would be destroyed. Confidentiality was also ensured within a given institution, so the content of an interview with a given fellow was not disclosed to the faculty of the program, and vice versa. If they wished, they could receive the audio files of their own interviews. They were also informed that they could decline to participate in the study any time they wished, but no participants expressed a wish to do so. Since the number of programs which actually provide ID fellowship are relatively scarce in Japan (only 34.1% of accredited programs and 35.6% of collaborative programs according to our previous survey),^8^ with fewer female physicians than other nations (the lowest rank among Organization for Economic Co-operation and Development countries),^10^ we decided not to provide detailed demographics of the participants in the current study for confidentiality concerns. The Kobe University Graduate School of Medicine Ethics Committee reviewed and approved this study (No. 1120).

## Results

### Structural differences

The structures of ID programs in Japan appeared to have considerable variations, but can be largely divided into two groups. Some fellows belonged to divisions of different subspecialties such as pulmonary medicine, had inpatient duty, and saw patients of their specialty while seeing infectious diseases occasionally. We here name it “type 1” structure, which is “The organ oriented style traditionally adopted in Japan”. Their institutions often do not even possess an ID department, while providing ID fellowship programs. In this case, both fellows and faculty physicians had only a small portion of time and effort devoted to infectious diseases, partly because they have so many other duties. In addition, it was surmised by comments that they tended to see particular infections related to their subspecialties; e.g. respiratory infections by pulmonologists, or infectious complications of hematological malignancies by hematologists. However, since some institutions lack the range of subspecialties to be covered, fellows may see infections in organs which are different from their original subspecialties cover.

“I belong to the Department of Pulmonary Medicine. Since I take care of respiratory illness, where there are many infections such as pneumonia, and since we have only a small number of internists, I take care of other infections broadly while being a member of Pulmonary Medicine, and I consider this a benefit of being here.” [Fellow 2]

“I take care of patients in an inpatient setting. Since I belong to Pulmonary Medicine, I mainly see pneumonia.” [Fellow 7]

“(My faculty) is pediatrics and does ID work as well. In fact, the faculty does many other things too.” [Fellow 1]

“In our hospital, we see a lot of infections. Our department mainly specializes in collagen vascular diseases and renal diseases, we see infections since we are pediatricians. I teach fellows as we see a variety of cases. We do not take care of adults, since one of our internists is an ID specialist, who is a pulmonologist.” [Consultant 2]

 “Maybe, 5 to 10 % of our patients do have infections. Currently most do not have collagen vascular diseases; they have hematological disorders with infections. Well, we call ourselves ‘Internal Medicine 1’.” [Consultant 5]

“Internal Medicine 1” refers to a classic department style of Japanese University Hospitals; i.e., one professor being at the top of the department and has various divisions of different subspecialties. For example, Department of Internal Medicine 1 might consist of a hematology division, endocrinology division, and rheumatology division.

On the other hand, some institutions do have ID departments. ID physicians see a variety of infections, without having specific organs of specific subspecialties.  In this case, some fellows see patients mainly as consultants and on an outpatient basis. They may or may not have inpatient duties. We here name this “type 2” structure, which is “Infection oriented style adopted mainly in North American countries”.

“We have an American style Department, our fellows do not take care of inpatients, we do pre-rounds in the morning, see new patients, and do formal rounds with faculties in the afternoon.” [Consultant 9]

“One takes care of outpatients, and the other takes consults and sees inpatients.” [Consultant 3]

“Rather than having an inpatient service, on top of being on call, we rather decided to do consultations mainly, and I believe this is basically the right thing to do.” [Consultant 7]

### Proportion, and “depth” of ID education

Time spent by the faculty physicians for fellows appeared varied among programs, ranging from full time level education, weekly meetings, to sporadic educational opportunities only when the fellow was in trouble. Some even do not have regularly held rounds.

“Fellows do an outpatient service once a week, and call the faculties if needed.” [Consultant 6]

“We pick up patients with infections at rounds held weekly, and teach on the diagnosis and treatment. Also, we ask fellows to help us about nosocomial infections or some procedures.” [Consultant 8]

“We sometimes see patients together and teach fellows directly, and also sometimes get calls from fellows about difficult to manage infections.” [Faculty 5]

“Probably, we do teach for one hour a day or so, such as doing evening rounds for example, or doing rounds together as a part of the Infection Control Team rounds. Or, sometimes we ask him to join the evening rounds of Surgery.” [Fellow 7, and the faculty belongs to Department of Surgery]

“Regular conferences are held every Monday, mainly held by ward pharmacologists. We also do rounds every Thursday.” [Consultant 1]

“We do not have formal training. They learn through experience, seeing both inpatients and outpatients.” [Fellow 8]

“I think the biggest problem is that we do not have rounds at all.” [Fellow 2]

### Segmental topics related to ID

Exposure to variety of subjects on ID also appears varied among institutions. For HIV/AIDS, some institutions do not see these patients at all, or fellows may not have the opportunity to care for these patients because other physicians exclusively see them.

“We basically do not see HIV at all.” [Consultant 7]

“So far, I have never seen a HIV patient.” [Fellow 2]

“Our hospital is not a “Kyoten Byoin” for HIV, so some hematologists personally see HIV a bit, but...” [Fellow 1]

“Kyoten Byoin” refers to institutions specifically arranged for HIV/AIDS care, designated by the Ministry of Health, Labor, and Welfare of Japan.

“We recently had a case of HIV in the outpatient setting, but we sent the patient to a University Hospital.” [Fellow 7]

“We see HIV patients, say about 20 to 30, but mostly they are seen by our professor in the outpatient setting.” [Fellow 5]

 “Two patients had positive HIV tests in the past, and one of them is followed by our faculty, the other was sent to another hospital.” [Fellow 4]

Some fellows, however, have the opportunity for HIV/AIDS care.

“HIV patients are mainly seen by the faculties, and I see only about 5 HIV patients.” [Fellow 9]

“I think it is good to see “big” infections such as HIV, malaria, or TB broadly.” [Consultant 3]

The attitudes toward travel or tropical medicine also varied among programs.

“We rarely see imported infections, to be honest.” [Fellow 1]

“As far as I know, we do not have doctors specializing in imported infections.” [Fellow 3]

“We see students at our University coming back from Southeast Asia who have got malaria or others, say, like once or twice a year, and these are all the imported infections we see.” [Fellow 5]

“We do see imported infections in the post-travel outpatient setting, but they are few in number.” [Consultant 9]

 Some programs had an exceptionally large volume of exposure to these patients.

“We usually see 10 imported infections in the inpatient setting at one time. Well, it depends on the season. Tropical diseases increase tremendously in the summer and spring vacation seasons, mostly Dengue this year. At other times, we usually see common infections.” [Consultant 3]

“We see about 20 to 30 malaria cases a year, some variation exists depending on the year.” [Consultant 3]

 Electives at other institutions domestically or abroad might overcome the problems of volume. However, this option did not appear common in most programs.

“For ID, we do not have any specific away elective program.” [Fellow 8]

“We have little away elective.” [Fellow 5]

Again, some exception exists.

“We can go to Thailand or African nations for 3 months as an option.” [Consultant 3]

### Infection control vs ID management

In Japan, hospital deans may require ID physicians to act as Infection Control Practitioners.

“We are obliged to do Infection Control work. We are viewed as having knowledge about infections, and this standpoints fits to the needs of the hospital; i.e. Infection Control.” [Fellow 10]

“Infection Control Team rounds are held once a week. We visit predetermined wards then, spending about 2 hours.” [Fellow 5]

“I think there is a gap between what we want to do and what the hospital wants us to do. I think that ID specialists should be adaptable, being able to do Infection Control as well, and this will help patients and the country as a whole. I think it is good to have Infection Control in the curriculum of ID fellowships, which will make Japanese ID programs somewhat special, and it meets the reality of the hospitals.” [Consultant 9]

“Our department has double structures, clinical ID and Infection Control. I think we are doing both, trying to have a good balance.” [Consultant 6]

### Board certification system

For questions regarding Board Certification provided by JAID, it appeared the attitudes toward the system are polarized to two extremes; one of complacency and the other of strong need for reform.

“For the system per se, I have never thought about it seriously.” [Fellow 10]

“I think it is fairly working.” [Consultant 6]

“Well, it is quite strict, I guess it is OK.” [Consultant 2]

“Well, I don’t have any opinion about it, really.” [Fellow 9]

“Oh, I have never thought about it... Sorry... Never thought about it, even though I am the division chief... Sorry. I cannot get around to that matter.” [Consultant 8]

“Well, sorry... I have never thought about it specifically... I cannot come up with any ideas now.” [Fellow 8]

Other opinions include,

“There are only small number of “Shidoi”, and so are the number of accredited programs. We happened to have an faculty member who is a “Shidoi”, so I am now trained in this accredited institution, but honestly, I think we should have more hospitals which provide ID fellowships.” [Fellow 2]

“I think we have so many ID related academic societies, like The Japanese Society of Chemotherapy, and I think there are several more. I guess they should be more unified. We have so many qualifications now.” [Fellow 2]

“You know, we have no specific benefits after becoming board certified.” [Fellow 5]

There was discussion about the Board Certification System related to the large variation of ID postgraduate educational programs.

“I guess there are huge variations among institutions. Now, even if you became board certified, well, what to say, you will have so much difference in experience, competency and so on.” [Fellow 8]

That variation might have caused differences in clinical competencies.

“I think that Japanese ID physicians lack competency, such as diagnostic ability, for example, like the ability to comprehend the patients after really seeing them.” [Fellow 8]

 Some expressed that the requirements of the JAID were too easy, which might hinder the quality assurance of ID fellows and their training. This potentially allows more physicians become to ID specialists, at the cost of lower quality.

“When you try to be board certified, there is a lack of cases... I think the board certification to become a rheumatologist is far more difficult. To become an ID specialist, you just send simple case summaries, passing the exam, and that is it. I think you have to make the ID board certification far more difficult to obtain, like the one in rheumatology, mandating actual fellowships, you have to be tougher on this, I think.” [Fellow 5]

“There are so many programs... I think the qualification criteria should be stricter. We should have fewer programs too.” [Fellow 7]

“We have Departments of Infection Control nationwide, and there are certain needs for Infection Control, but the need for a Department of Clinical ID is not that big, not to the level of needs for emergency medicine, sorry to say that. I think unless ID physicians possess a certain high quality, there will be no increase in need for them. I think we should improve the quality of ID specialists further. It may be difficult to improve and maintain the quality of ID, since many have different backgrounds, some are pediatricians, some are internists, some are surgeons; totally different. Unifying these would be very difficult, but still, I think we should improve the quality of the board exam.” [Consultant 7]

“I think the JAID system is lax, I mean the board certification. Unlike the United States, we do not have an equivalent organization to the ACGME, so quality control of the programs is difficult. Therefore, many hospitals can participate in ID fellowships. We often see cases in Japan where there is an ID program but the faculties are committed to totally different things. There are many ID programs but they are not really providing the fellowship. I think the content of the programs should be checked more, so that the quality is assured. Otherwise, there is no meaning in becoming an ID specialist, engendering many pseudo-specialists, this is I think the most important point.” [Consultant 9]

The opposite opinion was also expressed by some.

“I think we should restrict the number of Infection Control Doctor, but also should increase the number of ID specialists.” [Consultant 7]

Here “Infection Control Doctor” refers to physicians certified by the Japanese College of Infection Control. This qualification has its own criticism, since one can be certified by several lectures and paper works, without actual training or examination.^11^

“In Japan, we have not trained that many specialists, and I think we are making specialists with quality. But we do not have many Departments of ID to begin with. Many people who take the board exam belong to other specialties taking care of disorders of specific organs. I do not think many who passed the exam really went through ID training, and this is a problem in my opinion. However, if you make this more difficult, we will have fewer ID specialists, and this is far more problematic.” [Consultant 6]        

### Quality of “Shidoi”

“Shidoi” is a peculiar system built in Japan. However, there is no definition or mission of “Shidoi” concretely documented.

“I think the faculties should have enough knowledge and experience. They should be good as doctors... Sorry about my abstract explanation... But good as doctors. They should be able to see infections and diseases in organs different from their original specialties. They should act cross-sectionally, seeing patients at any ward in a hospital. For this, “Shidoi” should also be attractive as human beings too.” [Consultant 4]

“I feel strongly that the “Shidoi” system is now a mere façade. Even those who do not teach, and do not have experience in teaching can be “Shidoi”. There is no quality assurance about the actual content of teaching, and I think this is a huge problem.” [Consultant 3]

## Discussion

Little research addresses the actual status of specialty training in Japan. JAID provides a fellowship curriculum, only as a list of subjects. What percentage of these subjects need to be learned, and to what extent, is completely unknown. For ID specialization in the United States, The American Board of Internal Medicine (ABIM) requires fellows to become competent in: (1) patient care and procedural skills, (2) medical knowledge, (3) practice-based learning and improvement, (4) interpersonal and communication skills, (5) professionalism and (6) systems-based practice.12 Each infectious diseases training program must have a specific program director, who must spend at least 50% of their time in the educational program of the fellowship, and must be certified by both the infectious disease and general internal medicine (or pediatrics) boards. There are strict policy guidelines on supervision by all infectious diseases faculty members at each training base, and on clinical and research career development activities for all faculty members involved in fellowship training. Each infectious diseases fellow must complete at least 2 years of training, and the fellows must receive specific training in infection control, HIV/AIDS, microbiology (including laboratory work), sexually transmitted diseases, care of immunocompromised patients, and other subjects listed in the guidelines,12 although the US training tends to lack adequate training in infection control.^13^  The British curriculum for Infectious Diseases specialty is even more comprehensive and detailed, with a separate program specifically designed for Tropical Medicine.^14^ No such rigorous requirements exist in ID programs in Japan, and this fact has cast a cloud on the quality of ID physicians in Japan.

Our study suggested that there are largely 2 kinds of ID programs for postgraduate training in Japan. The first is the traditional, classic type 1 style traditionally adopted in Japan, where one belongs to some specialty other than ID, and learns Infectious Diseases, as a subcategory of that specialty. The other is more like a North American style ID program run by an ID department (type 2), where no specific organ is emphasized and they may not have inpatient duty, in order to experience many cases as specialists. Our study is a qualitative investigation so we were not able to measure which style is more popular quantitatively, but surmising comments from the participants suggests that the former is more likely to be popular in Japan. In other words, Infectious Diseases as a subspecialty is not yet fully recognized in Japan. Rather, the ID specialty in Japan is more like a subcategory of each subspecialty. In a previous survey about Postgraduate Training in Infectious Diseases, only 4 countries among 33 countries that completed questionnaires did not have a recognized Infectious Diseases subspecialty.1 Even though Japan has Infectious Diseases as a subspecialty on paper, it may not be so in a real sense.

The amount and the quality of teaching by faculty physicians appeared to differ considerably among programs. Some programs do have daily teaching rounds while others do not have any official teaching sessions. Again, JAID does not have any concrete regulations in regards to the role and effort of faculty physicians.

Opinions on the regulations and requirements by JAID appear also to differ among the participants. It may appear that those who are not committed to ID as subspecialty tended to be satisfied with current regulations, while those who are committed are not. Satisfaction is like a container, where a small container becomes full with a small amount of fluid while a bigger container will not.

There are several limitations to our study. First, as with any qualitative study, our findings are based on the personal perceptions of participants, and may not reflect generalizable facts. Since we did not conduct quantitative analyses, the findings are more for theory building, rather than for theory proof. Second, although we tried to elaborate our theory based on data provided, to the point there was little to add onto our theory, it is still possible that our analysis was not fully analyzed and some improvement might be attainable. Third, since we were not able to analyze all ID training of every nation, we are not perfectly sure whether some problems in Japan we described are specifically unique in Japan, or they are rather universal phenomena and many countries do have similar issues.

## Conclusions

Our qualitative study suggests that there may be significant variation in the content, the quality, and the concept of the programs among postgraduate ID fellowships in Japan. The perceptions by interviewees on the educational system also appears to differ, depending on the standpoints they have on ID physicians. There probably needs to be a coherency in the provision of ID fellowship programs so that fellows acquire competency in the subspecialty with sufficient expertise to act as independent ID specialists. Further studies are necessary for the improvement of ID subspecialty training in Japan.

### Acknowledgements

We appreciate Daniel J Mosby for his correction of English language.

### Conflict of Interest

The authors declare that they have no conflict of interest.

## Supplementary material

Supplementary file 1 Questionnaire used for the semi-structured interviews
