# Supplementary material for: A qualitative study of infectious diseases fellowships in Japan
Source: Int J Med Educ. 2016 Feb 21;7:62–8. doi: 10.5116/ijme.56b5.010c (PMC4764247; doi:10.5116/ijme.56b5.010c)
Supplement: Supplementary file 1 — Questionnaire used for the semi-structured interviews [file ijme-7-62-S1.pdf]

Appendix

Questionnaire used for the semi-structured interviews

For fellows

1. Age, sex, and previous training experience before enrolling in the current program, and reasons for entering into the current program.
2. What kind of training are you receiving currently?
3. What are advantages of the current program? What was the same as expected, and what was better than expected before joining it?
4. What are the disadvantages of the current program? What is missing and what should be done about ~~it~~?
5. What do you think of the current training system?
6. What do you think future ID training should be like?
7. What is your future career path you are dreaming of?
8. What kind of concerns do you have in your career?

For faculties

1. Age, sex, and specialties other than ID.
2. What kind of training are you providing to fellows currently?
3. What are the advantages of the current program? What kind of things do you think are important for the training?
4. What are the disadvantages of the current program? What is missing and what should be done about it?
5. What do you think of the current training system?
6. What do you think future ID training should be?
7. What kind of "Shidoi" is an ideal one? What do you think of yourself as a "Shidoi"?
